# Validation of the global activity limitation indicator in Taiwan

**DOI:** 10.1186/s12874-019-0693-0

**Published:** 2019-03-07

**Authors:** Ru-Ling Hsiao, Chih-Hsun Wu, Che-Wei Hsu, Yasuhiko Saito, Yu-Hsuan Lin

**Affiliations:** 1 Health Promotion Administration, Ministry of Health and Welfare, Taipei, Taiwan; 2 Department of Psychology, National Chengchi University, No.64, Sec.2, ZhiNan Road, Taipei, Taiwan 11605; 3 Population Research Institute, Nihon University, Tokyo, Japan

**Keywords:** Depressive symptoms, Functional health, Global activity limitation Indicator (GALI), Taiwan longitudinal survey on aging (TLSA)

## Abstract

**Background:**

The Global Activity Limitation Indicator (GALI) is a single-item measure of functional decline, it is widely used in Europe but it has never been validated in an Asian population. The aim of this study was to validate the GALI in a sample of older Taiwanese people and to explore whether it captured not only physical but also psychological limitations.

**Methods:**

Data for 4961 individuals (mean age, 62.4 ± 9.4 years; 47.2% men) were obtained from a national representative refresh cohort of the 8th wave of the Taiwan Longitudinal Survey on Aging. Logistic regression analysis was used to examine associations among the GALI, activities of daily living (ADLs) and instrumental activities of daily living (IADLs) and to explore whether depressive symptoms (measured by the Center for Epidemiologic Studies Depression Scale, CES-D) could be an indicator of reporting limitations on the GALI.

**Results:**

Responding to the GALI, 21.7% of the sample described themselves as ‘limited.’ In logistic regression, the GALI response was significantly associated with those who reported one or more ADL difficulties (odds ratio [OR] = 35.89, 95% confidence interval [CI] 21.10, 61.03) and IADL difficulties (OR = 13.37, 95%CI 10.09, 17.71), respectively. Furthermore, those with more depressive symptoms were more likely to report they were ‘limited’ on the GALI.

**Conclusions:**

These findings provided evidence that the GALI is a valid tool to assess general limitations in an Asian population. Furthermore, it captured psychological limitations to some extent. There were variations between Taiwan and European countries (as has been previously reported between European countries). The reporting level in the GALI by the Taiwan population was comparatively lower than that in European countries, highlighting the need to embrace cultural differences and to use caution when comparing GALI results across countries.

## Background

‘Adding Health to Years’ is one of the declared primary goals of the World Health Organization in response to global aging and with the aim of reducing unhealthy life years while increasing longevity [1]. However, achieving this is challenging. After reviewing population aging theories with survey data from three cohorts, Robine and Michel [2] stated that the complex interactions made it difficult to forecast future population aging trends, such as increased or reduced levels of disability. They suggested the need for ‘world harmonization of functional measurements’ as one of the components to address this problem.

A potential solution could be the Global Activity Limitation Indicator (GALI) [3], developed by the European Union as a tool to monitor healthy life years. The GALI is a one-item instrument that measures disability explicitly by self-report through answering the question ‘For at least the past 6 months, to what extent have you been limited because of a health problem in activities people usually do?’ The GALI is an easy, straightforward question, with good reliability and validity. A systematic review [4] of 9 studies concluded that the GALI has a sufficient repeated-measures reliability in a period of about 20 days, a good concurrent validity that relates to other health variables, and a good predictive validity of future health outcomes. However, researchers remain concerned about the effect of cultural differences on responses.

The first transnational study to validate the GALI used data from the Survey of Health and Retirement in Europe (SHARE), obtained from 11 European countries [5]. The results found that the GALI responses showed significant relationships with those from other disability and function measures. Overall, individuals with one or more limitations to their activities of daily living (ADLs) had an odds ratio (OR) of 8.63 to report limitations on the GALI relative to those without an ADL limitation. Similar findings were found for limitations to instrumental activities of daily living (IADLs; OR = 6.01) such as weaker grip strength and slower walking speed. A meta-analysis [5] found no significant variations among the 11 countries in the odds ratios for reporting limitation on ADLs, grip strength and walking speed. The only significant variation related to IADLs was between Greece and Sweden. Thus, having one or more limitations in IADLs increased the odds of reporting a limitation on the GALI across all countries, although it may be higher in some countries than others.

The second transnational study to assess the validity of the GALI used data from the European Health Interview Survey (EHIS), which covered a broader range of age (15 years and above) and countries (14 European countries) [6]. The results confirmed that, in general, responses to the GALI were significantly associated with reported limitations to ADLs (OR = 15.4), IADLs (OR = 11.4) and physical function (OR = 6.7). However, there was statistically significant heterogeneity in the magnitude of the odds ratios among the countries for all three measures, which may be partly due to different survey methods used across countries [6]. The odds of reporting a limitation on the GALI for an individual reporting any number of difficulties with ADLs, relative to those reporting none, were more pronounced in some countries than in others. For example, Romania, France, and Hungary were at the higher end, whereas Slovenia and Malta had much lower ORs. Similarly, the odds ratios related to IADLs varied from 7.4 in Latvia to 26.3 in Slovakia and those related to physical function limitations ranged from 4.2 in Latvia to 11.7 in Poland.

Together, these results confirmed the GALI to be a good indicator of disability, but they also revealed that cultural differences affect to some degree how people understand and respond to the GALI question in different countries. Both study reports [5, 6] pointed out that the subjective, self-reported nature of the GALI could be one of the sources of the variation. In other words, an individual’s response to the GALI question may depend on the psychological process of how people read, understand, initiate responses, compare these with social or cultural norms and come up with their final response. This process is too complex to allow straightforward analysis of its components. However, we hypothesized that adding a psychological component, such as depressive symptoms, could be useful in exploring this issue.

The purpose of this study, therefore, was to use an Asian cohort, the Taiwan Longitudinal Survey on Aging (TLSA), to test the validity of the GALI in a non-European country. In addition, the study examined whether a psychological factor, such as the presence of depressive symptoms, can indicate the probability of reporting limitations based on the GALI.

## Methods

### Study design and participants

The TLSA is a population-based national representative longitudinal survey in Taiwan. The data used in the present study were from the refresh cohort of the 8th wave of the TLSA conducted in 2015. The target population of the refresh cohort were residents aged 50 and above at the end of April 2015, as recorded in Taiwan household registration data. A three-stage probability proportional to size method was used for sampling and the data were collected via at-home face-to-face structured interviews conducted by trained interviewers [7]. The study was approved by the Research Ethics Committee of the National Health Research Institutes, Taiwan (EC1040401-F), and a written informed consent was obtained from the respondents before the interview.

A total of 7500 residents were drawn, of which 5304 agreed to participate in the survey. The overall response rate was 70.7%. After excluding the data of 307 participants who answered via proxy and 36 participants who did not complete the relevant questionnaires, a total of 4961 participants were included in the analysis.

### Measures

The GALI is a single-item survey instrument that asks the question ‘For at least the past 6 months, to what extent have you been limited because of a health problem in activities people usually do?’ There are three possible responses: ‘severely limited’, ‘limited but not severely’ and ‘not limited at all’. The item and responses were translated from English to Traditional Chinese and then back-translated into English by a different bilingual speaker who had not participated in the forward-translation phase. The Traditional Chinese version of the GALI item was then showed to several older Taiwanese people to check if they could understand the question correctly and found it easy to respond to. We followed the coding method used in both European studies [5, 6]. The severe and moderate limitation categories were combined in this study into ‘limited.’

ADLs and IADLs were used to measure limitations to living independently. ADLs comprise the following six items: self-feeding, transferring, walking around the house, dressing, bathing and using the toilet. IADLs comprise eight items: managing money, grocery shopping, doing heavy housework, doing light housekeeping (such as washing dishes), using the phone, preparing meals, doing laundry and taking medicine. The participants were asked if they had difficulties performing these activities by themselves. For each item, ‘no difficulty’ was coded as 0 and any difficulty was coded as 1. Higher ADL and IADL scores reflected a poorer function of living independently.

The Center for Epidemiologic Studies Depression Scale (CES-D) was used to measure depressive symptoms in all waves of the TLSA and has been shown to be a reliable and valid measure of geriatric depressive symptoms among older adults in Taiwan [8]. The version of the CES-D used in the 8th wave of the TLSA comprises 11 items. For each item, the participants are asked how often during the past week they had felt as described (such as ‘I feel lonely’). The options are none (coded as 0), rarely (1), sometimes (2) and most of the time (3). The total score of the CES-D ranges from 0 to 33, with higher scores representing more severe depressive symptoms. In this study, we determined the cut-off point on the basis of a previous TLSA study [9] and divided participants into two groups. Those who scored 0–10 were categorized as the ‘low depressive symptom’ group, and those who scored 11 or higher were categorized as the ‘high depressive symptom’ group.

### Statistical analysis

All data were analyzed using SAS V.9.3 (SAS Institute, Cary, North Carolina, USA). We applied logistic regression analyses via PROC SURVEYLOGISTIC to investigate the relationships between the GALI and the two measures of disability (ADLs and IADLs) after adjusting for the effects of age. The probability of being classified as ‘limited’ or ‘not limited’ on the GALI was estimated for each category or value of the measured variable of interest. Odds ratios (ORs) were computed to present the strength of association between ADLs/IADLs and GALI in the logistic regression models, after adjusting for sex (male/female), age (measured in years), education level (elementary school or less vs. junior high school or above) and CES-D group (low vs. high depressive symptoms). All estimates and standard errors were weighted to adjust for unequal response rates by age, sex and county. A two-sided *p*-value <.05 was considered statistically significant.

## Results

A total of 4961 participants were included in the analysis. The mean age was 62.4 years; 47.2% were men. In answering the GALI, 21.7% reported limitation; 3.5% reported at least one difficulty with an ADL and 14.5% reported at least one difficulty with an IADL (Table 1).

**Table 1:** Characteristics of the population in TLSA (2015), Taiwan

	Taiwan^a^
Data collection year	2015
Sample size (N)	4961
Response rate (%)	66.1
Mean age (years)	62.4 ± 9.4
% Male	47.2
GALI (% limited)	21.7
ADL (% having difficulty with 1 or more activity)	3.5
IADL (% having difficulty with 1 or more activity)	14.5

TLSA, Taiwan Longitudinal Survey on Aging; GALI, Global Activity Limitation Indicator; ADL, activities of daily living; IADL, instrumental activities of daily living

^a^Results account for survey designs using sampling weights

**Fig. 1:**
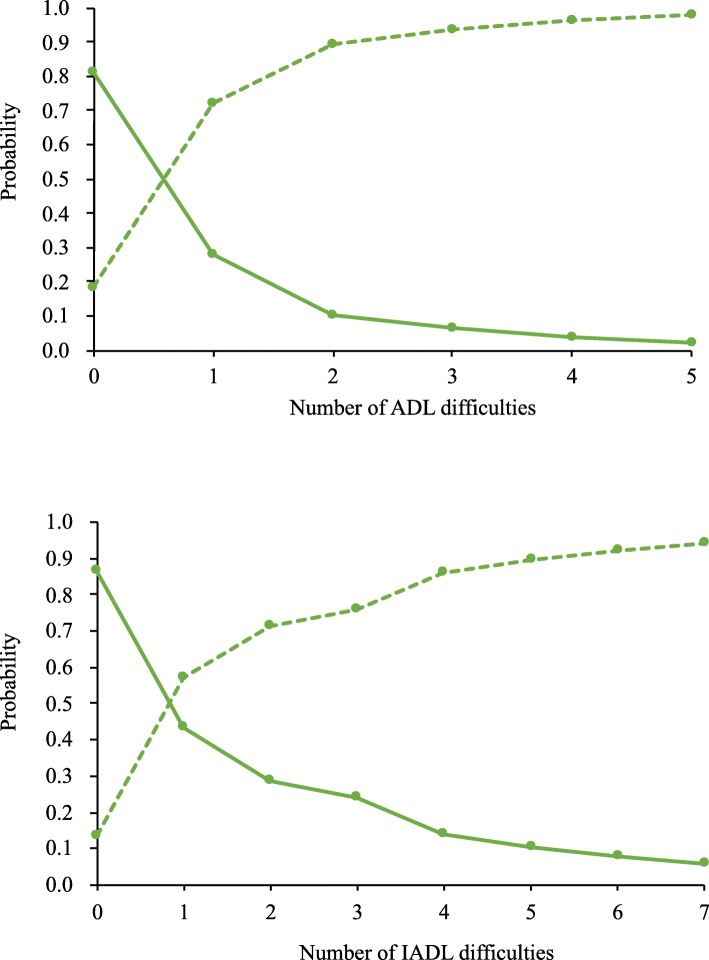
Probability distribution of the GALI against ADL/IADL difficulties in TLSA (2015), Taiwan

We used logistic regression models to examine whether responses to the GALI reflected overall disability according to the other measures. The overall association between the GALI and the other two disability measures showed that the GALI significantly reflected more severe limitations to ADLs and IADLs. For those who had at least one difficulty with an ADL or IADL, the probabilities that they would also report limitation on the GALI were 89 and 68%, respectively. Consequently, those who reported to have no difficulty with any ADL or IADL measures, the probabilities that they would report limitation on the GALI were 18 and 14%, respectively (Table 2). The ORs for reporting limitation based on the GALI, after adjusting for age and sex, for those who reported any difficulties on ADLs or IADLs relative to those reporting none were 35.89 (95%CI: 21.10, 61.03) for ADLs and 13.37 (95%CI: 10.09, 17.71) for IADLs. These results remained similar after further adjustment for level of education (Table 3). As the number of reported difficulties in ADLs and IADLs increased, the proportion reporting limitation based on the GALI also increased (Fig. 1). With one to five reported difficulties with ADLs, the probabilities of reporting limitation based on the GALI were 72, 90, 94, 96 and 98%, respectively. The trajectory of the increase seemed more linear for difficulties with IADLs: with one, three, five and seven reported difficulties with IADLs, the probabilities of reporting limitation on the GALI were 57, 76, 89 and 94%, respectively.

**Table 2:** Predicted probability of the GALI-defined activity limitations by ADL/IADL difficulties in TLSA (2015), Taiwan

		GALI	
		Not Limited	Limited
ADL difficulties	No	0.82	0.18
	1+	0.11	0.89
IADL difficulties	No	0.86	0.14
	1+	0.32	0.68

GALI, Global Activity Limitation Indicator; ADL, activities of daily living; IADL, instrumental activities of daily living; TLSA, Taiwan Longitudinal Survey on Aging

**Table 3:** Association between the GALI and ADL/IADL difficulties in TLSA (2015), Taiwan

	Model 1		Model 2		Model 3	
	OR	95% CI	OR	95% CI	OR	95% CI
Age	1.05	(1.04–1.06)	1.05	(1.04–1.06)	1.05	(1.04–1.06)
Sex	0.87	(0.75–1.01)	0.92	(0.78–1.07)	1.00	(0.85–1.17)
Education			1.39	(1.14–1.69)	1.35	(1.11–1.65)
ADL (1+ vs 0)	35.89	(21.10–61.03)	35.09	(20.52–60.00)	26.61	(15.49–45.70)
CES-D					4.53	(3.51–5.86)
Age	1.02	(1.01–1.03)	1.01	(1.00–1.02)	1.02	(1.00–1.03)
Sex	1.06	(0.90–1.25)	1.11	(0.94–1.32)	1.18	(1.00–1.39)
Education			1.43	(1.16–1.75)	1.40	(1.14–1.71)
IADL (1+ vs 0)	13.37	(10.09–17.71)	13.29	(10.03–17.61)	10.92	(8.17–14.60)
CES-D					3.46	(2.58–4.64)

Model 1 adjusted for age and sex (male/female)

Model 2 adjusted for age, sex (male/female) and education level (elementary school or less vs. junior high school or above)

Model 3 adjusted for age, sex (male/female), education level (elementary school or less vs. junior high school or above) and CES-D groups (low vs. high depressive symptoms)

GALI, Global Activity Limitation Indicator; ADL, activities of daily living; IADL, instrumental activities of daily living; TLSA, Taiwan Longitudinal Survey on Aging

We explored whether depressive symptoms, measured by CES-D, were a significant indicator for reporting limitation based on the GALI with ADLs or IADLs as covariates. After adjusting for age, sex and education, individuals in the ‘high depressive symptom’ group were more likely to report limitation based on the GALI than those in the ‘low depressive symptom’ group. The ORs were 4.53 (95%CI: 3.51, 5.86) and 3.46 (95%CI: 2.58, 4.64), with ADLs and IADLS as covariates, respectively, in the regression model (Model 3 in Table 3).

## Discussion

This study is one of the first studies to test the validity of the GALI in an Asian population. The results provided evidence that the GALI appeared to be a good indicator of disability in older adults, as found in previous studies. Our study also showed a stronger association between the GALI and ADLs, but a weaker association between the GALI and IADLs, which is consistent with the findings of the EHIS study [6].

We compared the results in Taiwan to both European studies. In this study, the percentage that reported limitation based on the GALI was 21.7%, which was lower compared to 11 European countries in the SHARE study, for which the lowest percentage was 30.4% (in Greece) [5]; however, it was higher than the percentages reported in Cyprus (18.1%) and Belgium (20.8%) in the EHIS study. The percentage that reported at least one ADL difficulty was 3.5%, which was also lower than in all 11 European countries in the SHARE study (lowest: Switzerland 6.8%) and only a little higher than France (3.4%) in the EHIS study. The percentage that reported at least one IADL difficulty was 14.5%, which was about the same as in Sweden (14.8%, fifth lowest in the SHARE study) and Spain (14.3%, sixth lowest in the EHIS study). Overall, the limitation profile for Taiwan according to these three measures was closest to those of Romania (22.5% on the GALI, 5.9% on ADLs, 12.3% on IADLs), France (25.2% on the GALI, 3.4% on ADLs, no IADLs data) and Cyprus (18.1% on the GALI, 5.3% on ADLs, 13.0 on IADLs) in the EHIS study.

In this study, the odds ratio for individuals with at least one difficulty in ADLs to report a limitation based on the GALI was 35.89 (95%CI: 21.10, 61.03) relative to those without any ADL difficulties. This was significantly higher than the overall results of the SHARE study (OR = 8.63, 95%CI: 7.13, 10.46) and the EHIS study (OR = 15.4, 95%CI: 11.7, 20.3). However, a comparison by country showed the odds ratio for Taiwan was close to those of Romania (OR = 53.2, 95%CI: 35.6, 79.6), France (OR = 47.7, 95%CI: 31.1, 73.2) and Hungary (OR = 28.4, 95%CI: 18.6, 43.4) in the EHIS study. In our study, the odds ratio for people who had one or more difficulties in IADLs to report limitation based on the GALI was 13.37 (95%CI: 10.09, 17.71) relative to those without IADL problems. This was significantly higher than the overall results of the SHARE study (OR = 6.01, 95%CI: 5.02, 7.21), but not the EHIS study (OR = 11.4, 95%CI: 9.7, 13.3). Moreover, considering that the odds ratios might have over-estimated the strength of the association, we used a Poisson regression approach [10] to calculate the prevalence ratios (PR). The PR for those with/without ADL limitations on reporting the GALI was 3.34 (95%CI: 2.99, 3.74), and 4.82 (95%CI: 4.29, 5.42) for those with/without IADL limitations. These results show that even when using a stricter estimation method, GALI is still significantly associated with ADL and IADL.

Both TLSA and SHARE focused on older populations (people aged 50 years and above), whereas EHIS included a more extensive age range (aged 15 years or more). Interestingly, the percentage of reporting limitations based on the GALI, the ADLs, and the IADLs in this TLSA study (Table 1) was lower than those in the SHARE study but somewhat more similar to those in the EHIS study. This may be attributable to the two issues raised by Berger et al. [6]: comparability of the data and cultural differences in self-reporting. With the caveat that the comparability of data might be compromised by the different methods used across surveys, we further investigated the demographics of the sample.

The percentage of men in the TLSA sample (47.2%) was within the range for the countries in the SHARE study (42.1 to 47.4%). The mean age of the TLSA participants (62.4 ± 9.4 years) was a little younger than those of the 11 countries in the SHARE study, with mean ages ranging from 63.9 ± 9.7 years to 66.9 ± 10.6 years. Although this difference was quite small, we tested the effect of age by selecting the participants aged 65 years or more and ran the analyses again. The selected sample had a mean age of 73.3 ± 6.5 years; 34.3% reported a limitation based on the GALI, 7.3% reported at least one ADL difficulty and 30.5% reported at least one IADL difficulty. After adjusting for age and sex, the odds ratios for reporting limitation based on the GALI by individuals with at least one difficulty with ADLs or with IADLs were 22.27 (95%CI: 11.98, 41.38) and 8.55 (95%CI: 6.14, 11.90), respectively, relative to those without difficulties. Compared to the results for the full sample, the results for this selected older sample seemed closer to those of the SHARE study; this supports the commonly observed age effect of a positive correlation between older age and functional decline.

Subjectivity is a potential source of variability in answers to the GALI. The aim of the GALI is to provide a harmonized functional measurement, so it is important to take into account the types of situation in which individuals report limitation based on the GALI. For instance, it would be beneficial to know which nationalities were more likely to be hesitant to report limitation based on the GALI; however, establishing this for each country would be complex and time-consuming. A more efficient approach might be to identify what the GALI captures. In this and other studies [5, 6], individuals with ADL or IADL difficulties were more likely to report limitation based on the GALI, as were those with weaker grip strength or slower walking speed. These results imply that the GALI measures physical limitations; however, it may not measure physical limitations alone. In the present study, people in the ‘high depressive symptoms’ group were about 4.53 times (with ADLs as covariate) and 3.46 times (with IADLS as covariate) more likely to report limitation based on the GALI than those in the ‘low depressive symptom’ group.

Sensitivity tests also found that the CES-D was still a good indicator for the GALI in the subpopulation with no ADL/IADL limitations. People in the high CES-D group were more likely to report limitation based on the GALI than those in the low CES-D group: in the subpopulation without ADL limitations (OR: 4.53, 95%CI: 3.49, 5.87), without IADL limitations (OR: 3.56, 95%CI: 2.51, 5.04), and without both ADL and IADL limitations (OR: 3.61, 95%CI: 2.53, 5.14). Furthermore, among those without ADL difficulties, the CES-D score was significantly higher for those who reported limitation based on the GALI (6.11 ± 6.19) than for those who did not (2.95 ± 3.98). The results were similar for those without IADL difficulties: the CES-D score was significantly higher for individuals who reported limitation based on the GALI (5.21 ± 5.70) than for those who did not (2.86 ± 3.85). These results suggest that the GALI also captures psychological limitations, even without the presence of ADL or IADL limitations. A study in Europe also showed a relationship between GALI responses and mental morbidity [11].

Another issue with data comparability is whether responses answered by proxy should be included or excluded in the analysis. The SHARE and EHIS studies [5, 6] both included proxy data, but proxy data were not included in the present analyses (concerning the subjectivity of the CES-D). We investigated the data answered via proxy. For the 307 responses answered by proxy, the mean age was 76.7 years and 48.9% were men. The percentage that reported limitation based on the GALI was 88.9%, with 70.6% reporting at least one ADL difficulty and 94.9% reporting at least one IADL difficulty. This indicated that the individuals with proxy responses were generally more limited than those in the main study population. Meanwhile, the inclusion of proxy data in the analyses produced a total sample size of 5289 participants (excluding 15 participants with missing ADL, IADL, or education level data); mean age was 63.1 years and 47.3% were men. In this larger sample, 25.2% reported limitation based on the GALI and 6.9 and 18.6% reported at least one difficulty with ADLs or IADLs, respectively. The results of the logistic regression model analyses showed that individuals with at least one ADL or IADL difficulty had probabilities of 91 and 73%, respectively, of reporting limitation based on the GALI. For those with neither ADLs nor IADLs problems, the probability that they would report limitation based on the GALI were 20 and 14%, respectively. After adjusting for age and sex, the odds ratios for reporting limitation based on the GALI for those who had difficulties with one or more ADLs or with one or more IADLs (relative to those reporting no difficulties) were 40.21 (95%CI: 25.17, 64.25) and 16.60 (95%CI: 12.71, 21.69), respectively. These results showed that even after including the data by proxy respondents, the percentage of those with GALI limitations was still lower than in all 11 European countries in the SHARE study, and the odds ratios to report limitation based on the GALI among those with any difficulties on ADLs or IADLs were still considerably higher than for the overall results of the SHARE study. This suggests that excluding the data from proxy responses in the present analyses did not have much impact on the results.

The GALI was not included in the TLSA survey until the 8th wave. Because of this, a major limitation of the present study was the lack of longitudinal data to validate the use of the GALI through exploring period and cohort effects. A study based on four waves of SHARE data from 2004 to 2013 found that the estimated trends of disability differed across countries and the measures used [12]. These findings suggested that the GALI could be a useful tool in comparing disability trends between and across countries. A study that investigated trends in later life disability analyzed the six waves of TLSA data from 1989 to 2007 (which did not include the GALI) and also found measurement differences [13]. The study noted that the trend for ADL limitation was flat, whereas limitations related to IADLs, particularly, seeing and hearing declined substantially. The authors suggested that these improvements in functional limitation in Taiwanese elderly may have been related to the substantial increase in level of education. However, the findings also raised the question of whether this was a period effect that reflected the rapid development of Taiwan during the past three decades or a reflection of a global trend. A worldwide harmonized functional measure could be helpful to address such questions.

## Conclusions

The findings of this study provided support for the GALI as a valid tool to measure general limitations in an Asian population, such as Taiwan. Furthermore, the GALI may not only reflect physical limitations but may also capture psychological limitations to some extent. Thus, the GALI may ‘assess more with less items,’ in accordance with Verbrugge’s criterion for a good disability measure [14]. There were differences between the results for Taiwan and those for European countries. These results highlight the need to recognize cultural differences and to use caution when comparing GALI results between and across countries.

## Abbreviations

ADLs: Activities of daily living
CES-D: Center for Epidemiologic Studies Depression Scale
EHIS: European Health Interview Survey
GALI: Global Activity Limitation Indicator
IADLs: Instrumental activities of daily living
SHARE: Survey of Health and Retirement in Europe;
TLSA: Taiwan Longitudinal Survey on Aging
